# Human Activity as a Growing Threat to Marine Ecosystems: Plastic and Temperature Effects on the Sponge *Sarcotragus spinosulus*

**DOI:** 10.3390/toxics13010066

**Published:** 2025-01-20

**Authors:** Jessica Lombardo, Maria del Mar Ribas-Taberner, Maria Magdalena Quetglas-Llabrés, Samuel Pinya, Llorenç Gil, Silvia Tejada, Antoni Sureda, Montserrat Compa

**Affiliations:** 1 Research Group in Community Nutrition and Oxidative Stress (NUCOX), University of Balearic Islands, 07122 Palma de Mallorca, Spain; jessica.lombargo@uib.cat (J.L.); m.ribas@uib.cat (M.d.M.R.-T.); m.quetglas@uib.cat (M.M.Q.-L.); montserrat.compa@uib.es (M.C.); 2 Health Research Institute of Balearic Islands (IdISBa), 07120 Palma de Mallorca, Spain; s.pinya@uib.es (S.P.); silvia.tejada@uib.es (S.T.); 3 Interdisciplinary Ecology Group, University of the Balearic Islands, 07122 Palma de Mallorca, Spain; lorenzo.gil@uib.es; 4 Laboratory of Neurophysiology, University of the Balearic Islands, 07122 Palma de Mallorca, Spain; 5 CIBEROBN (Physiopathology of Obesity and Nutrition), Instituto de Salud Carlos III, 28029 Madrid, Spain

**Keywords:** temperature, plastics, stress, sponge, *Vibrio*, Balearic Islands

## Abstract

Human activities increasingly threaten marine ecosystems through rising waste and temperatures. This study investigated the role of plastics as vectors for *Vibrio* bacteria and the effects of temperature on the marine sponge *Sarcotragus spinosulus*. Samples of plastics and sponges were collected during July, August (high-temperature period), and November (lower-temperature period). Bacterial growth and sponge responses were analysed using biochemical biomarkers. The results revealed a peak in colony-forming units (CFU), particularly of *Vibrio alginolyticus*, on plastics and sponges in August, followed by a decrease in November. In August, CFU counts of *Vibrio* spp. were significantly higher in sponges with poor external appearance (characterized by dull coloration and heavy epiphytic growth) but returned to levels observed in healthy sponges by November. Microplastics were detected in the tissues of both sponge groups, with higher concentrations found in affected specimens. Biomarker analyses revealed increased lysozyme, glutathione S-transferase, catalase, and superoxide dismutase activities in healthy sponges during August, while malondialdehyde levels, indicating oxidative damage, were higher in affected sponges. In conclusion, affected sponges exhibited elevated CFU counts of *Vibrio* spp. and reduced antioxidant and detoxification responses under elevated temperatures. These findings suggest that combined impacts of plastics and warming may pose significant risks to *S. spinosulus* in the context of global climate change.

## 1. Introduction

Human activities are increasingly impacting marine ecosystems, resulting in significant environmental degradation. Among the most concerning consequences of this anthropogenic influence are rising pollution levels, particularly plastic waste, and increasing global temperature [1,2]. These factors not only threaten the biodiversity of marine environments but also create favourable conditions for the proliferation of alien species and harmful pathogens, some of which may be invasive or even pathogenic [3].

Over the past few decades, global plastic production and consumption have surged, leading to an alarming accumulation of plastic debris in the world’s oceans and seas [4]. Plastic consumption increased approximately 180-fold between 1950 and 2018 [5]. Currently, global plastic production surpasses 400 million tons annually and is expected to grow exponentially in the coming years [6]. Each year, an estimated 8 to 10 million tons of plastic, including both macroplastics and microplastics (MPs), enter the oceans, accounting for 80% of all marine pollution [5]. Marine animals, ranging from vertebrates such as fish, turtles, and seabirds to invertebrates like sponges and cnidarians, are significantly affected by plastic pollution [7]. These plastic materials can entangle organisms, restricting their mobility and causing physical injuries [8]. Additionally, plastics are frequently ingested, either because they are mistaken for food or accidentally consumed. This ingestion can result in damage to the digestive tract, reduced appetite, impaired reproductive capacity, and even death [7,9]. Furthermore, plastic debris, particularly MPs, has become ubiquitous in marine environments, making it increasingly challenging for organisms to avoid or escape exposure. Beyond direct physical harm, plastics also act as vectors for microorganisms. Floating plastics in the oceans can accumulate bacteria, viruses, and other pathogens in polluted waters [10]. This phenomenon has raised concerns about the potential for plastics to act as carriers of infectious diseases, as microorganisms can survive on plastic surfaces for extended periods [11]. As plastics are transported across vast distances, they may facilitate the spread of pathogens to new and previously unexposed regions or increase the degree of exposure to existing microorganisms, exacerbating the threat to marine biodiversity and human health.

Another pressing issue is the rise in ocean temperatures, largely driven by climate change. The warming of marine environments can profoundly affect the distribution and behaviour of marine species [12]. Organisms exposed to temperature changes may be forced to migrate to cooler waters in response to rising temperatures. For sessile organisms, such as sponges, this inability to relocate can result in significant mortality. Increased water temperatures can disrupt physiological processes, including filtration, nutrient uptake, and symbiotic relationships with microorganisms [13]. These disruptions can weaken the sponges’ ability to adapt to environmental changes, increasing their vulnerability to disease and predation. Furthermore, prolonged thermal stress can lead to tissue necrosis, bleaching (in species that host photosynthetic symbionts), and eventually death [14]. Several documented cases of mass mortality events among sponges have been linked to marine heatwaves, during which elevated temperatures persisted over extended periods [15,16]. Recovery has been slow or entirely absent in some areas, with mortality rates continuing to worsen even after the surface heatwave subsided [17].

*Sarcotragus spinosulus* Schmidt, 1862, is a species of Demospongiae commonly found in shallow marine environments of the Mediterranean Sea and adjacent Atlantic regions [18]. This sponge plays a critical role in benthic ecosystems, contributing to nutrient cycling through its efficient filtration capacity. It is characterized by a robust, irregularly lobed body structure, and a complex internal canal system. Despite its ecological importance, *S. spinosulus* is particularly vulnerable to environmental stressors, including pollution, invasive species, habitat destruction, and rising sea temperatures [19]. Furthermore, prolonged thermal stress has been associated with cases of tissue necrosis and mass mortality events [20,21]. Additionally, its slow growth rate and susceptibility to pathogenic outbreaks further complicate recovery efforts, highlighting the need for conservation measures to protect its populations.

**Figure 1:**
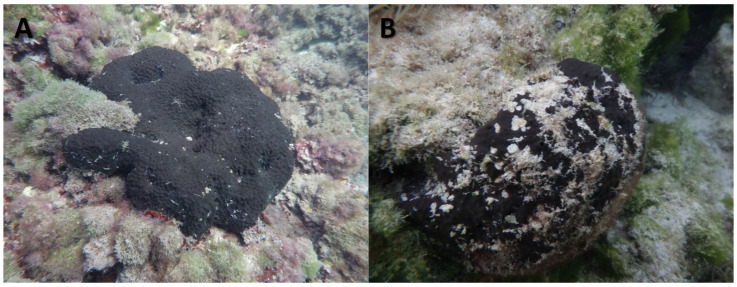
Representative images of (**A**) healthy and (**B**) affected specimens of *Sarcotragus spinosulus*.

In 2022 and 2023, several heatwave events were recorded in the Balearic Islands region, with temperature peaks exceeding 30 °C, particularly in August [22,23]. During the hottest months, noticeable changes in some *S. spinosulus* specimens were observed (personal observations). These changes included a shift to duller coloration and a significant increase in epiphyte growth (Figure 1). These changes suggest a state of stress or weakness in the affected individuals.

Thus, the aim of this study was to investigate the combined effects of plastic pollution (also as potential carriers of pathogens) and rising ocean temperatures on the sponge *S. spinosulus*, evaluating the presence of several *Vibrio* species of the sponge surface and the intake of MPs, and some biomarkers of oxidative stress, detoxification, and immune response.

## 2. Materials and Methods

### 2.1. Study Area

**Figure 2:**
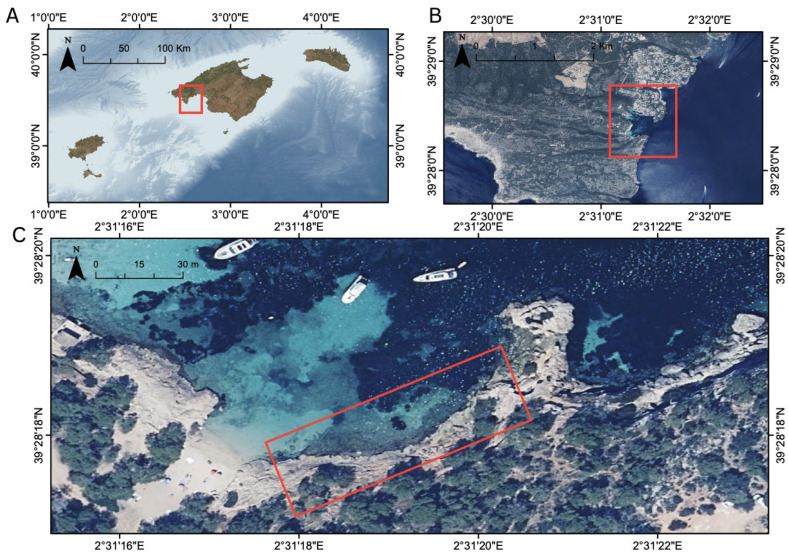
Geographic location of the study area for Portals Vells in the Balearic Islands, western Mediterranean Sea: (**A**) Balearic archipelago showing Mallorca, Menorca, Ibiza, and Formentera islands; (**B**) detailed view of study area on the southwest coast on the island of Mallorca (red rectangle); (**C**) aerial photograph of the study site showing the sampling area (red rectangle).

The study area is located in the Portals Vells area, on the southwest coast of Mallorca Island, part of the Balearic Islands in the western Mediterranean Sea (Figure 2). The beach is exposed to east and southeast winds, which facilitate the accumulation of debris, including plastics, in the shallower areas. This beach is heavily frequented by tourists, particularly during the summer months (July and August), and is a popular spot for boat anchoring. Just south of Portals Vells lies the Marine Area of Cap de Cala Figuera (ES5310103), designated as a Site of Community Interest. This area harbours *Posidonia oceanica* seagrass meadows, which play a critical role in carbon fixation and storage. Additionally, it supports the gastropod *Dendropoma lebeche*, a protected endemic species of the Mediterranean Sea.

### 2.2. Sample Collection

All samples were collected through free diving near the shoreline at an average depth of 0.5–1 m on the western side of the beach. During an initial sampling in the first week of July 2024, a total of 15 samples of *S. spinosulus* were obtained from different specimens, each approximately 1 cm^2^ in size. A second sampling in the third week of August yielded 10 samples of visually healthy sponges (brightly coloured and free of epiphytes) and 10 samples of affected sponges (duller coloration and abundant epiphytic growth). A third sampling in the first week of November involved collecting samples from the same 10 healthy and 10 affected sponges. At the collection site, the samples were carefully inspected, epiphytes were meticulously removed, and the outer surface of each sponge sample was swabbed using a sterile swab. Each swab was placed in a tube containing 1 mL of PBS and transported to the laboratory. Sponge samples were rapidly frozen in liquid nitrogen and stored at −80 °C until further analysis. Permission for the collection of *S. spinosulus* specimens was obtained from the Direcció General de Pesca (Balearic Government).

During each sampling event, 40 pieces of plastic debris, approximately 1 cm^2^ in size, were randomly collected. These plastics were processed in the same manner as the sponge samples and stored in tubes with PBS until subsequent laboratory analysis.

### 2.3. Seawater Temperature

Water temperature was measured during each sampling event using multiparameter equipment (YSI Pro30 and ProODO, Yellow Springs, OH, USA) at a depth of 0.8 m. Additionally, during the August sampling, temperature variation over a full day was monitored by deploying a HOBO^®^ Tidbit^®^ MX temperature sensor (Interworld Highway, LLC, Long Branch, NJ, USA) at a depth of 0.8 m.

### 2.4. Microbiological Analysis

To evaluate the microbiological presence and distribution of *Vibrio* species on *S. spinosulus* and plastic samples, a sterile inoculation loop was used to transfer material collected from the surface of each sample to CHROMagar *Vibrio* medium (CHROMagar Microbiology, Saint Denis, France). The medium (pH 9.0) contained the following ingredients per liter: 15.0 g agar, 8.0 g peptone and yeast extract, 51.4 g salts, and 0.3 g chromogenic mix. This medium is a highly selective chromogenic agar designed for the isolation and differentiation of *Vibrio* species. The preparation of the medium followed the manufacturer’s detailed instructions, ensuring optimal conditions for species-specific chromogenic reactions.

The inoculated plates were incubated at 37 °C in the dark for 24 h according to the manufacturer’s recommendation. The medium allows these organisms to be distinguished from each other based on a proprietary chromogenic reaction. Following the incubation period, all plates were examined for growth and the formation of the colony-forming units (CFUs) were manually counted: *Vibrio alginolyticus* (cream-colored colonies), *Vibrio vulnificus* (green-blue colonies), and *Vibrio parahaemolyticus* (mauve-purple colonies).

### 2.5. Plastic Analysis and Characterization

For the analysis of MPs in *S. spinosulus*, a subsample from each sponge collected in August was transferred to a labelled glass Erlenmeyer flask and incubated in a saline solution (1 L H_2_O with 120 g NaCl) combined with a 10% potassium hydroxide (KOH) solution at a ratio of 20 mL KOH per gram of tissue. Both solutions were pre-filtered to maintain purity. The chemical digestion process lasted 48–72 h at 60 °C, with the flasks covered in aluminium foil to prevent airborne contamination. Following digestion, the resulting mixture was filtered using a vacuum filtration apparatus. Filtration was carried out under a fume hood using BRANCHIA microfiber glass paper filters (grade BGF-3, 47 mm diameter, 1.2 µm pore size) to reduce exposure to airborne contaminants. The filters were then carefully transferred into individual glass Petri dishes and allowed to dry at room temperature for 24 h [24]. Once dried, the filters were analysed under a Leica EZ4 stereomicroscope for visual identification of MPs. MPs were counted for each specimen, and their colours and shapes were documented. Shapes were classified as either fibres or fragments. High-resolution images of the MPs were captured with a Leica DFC295 digital camera, offering optical magnification up to 5×, and processed using the Leica Application Suite software v4.

To analyse MPs in *S. spinosulus*, a subsample from each sponge collected in August was transferred to a labelled glass Erlenmeyer flask and incubated in a saline solution (1 L H_2_O with 120 g NaCl) combined with a 10% potassium hydroxide (KOH) solution at a ratio of 20 mL KOH per gram of tissue. Both solutions were pre-filtered to maintain purity. The chemical digestion process lasted 48–72 h at 60 °C, with flasks covered in aluminium foil to prevent airborne contamination. Following digestion, the mixture was filtered using a vacuum filtration apparatus under a fume hood. Filtration was performed using BRANCHIA microfiber glass paper filters (grade BGF-3, 47 mm diameter, 1.2 µm pore size) to minimize airborne contaminants. Filters were then carefully transferred into individual glass Petri dishes and dried at room temperature for 24 h [24].

To minimize contamination during field and laboratory work, all equipment was thoroughly rinsed three times with purified water from a Milli-Q^®^ A10 Direct Water Purification System (Merck KGaA, Darmstadt, Germany). Lab personnel wore cotton lab coats and nitrile gloves throughout the experimental procedures to reduce contamination risks. Additionally, all saline solutions were pre-filtered to eliminate potential MPs, and control samples using distilled water were inspected under a stereomicroscope to identify any airborne plastic contamination.

The polymeric composition of the 40 plastic samples collected in August was determined using micro-attenuated total reflection micro-Fourier-transform infrared spectroscopy (μ-ATR-FTIR) (Bruker, OPTICS, Mannheim, Germany). FTIR absorption spectra were acquired by averaging 250 scans within the mid-infrared range of 400–4000 cm^−1^. The resulting spectra were compared against commercial and in-house spectral libraries, requiring a minimum hit quality index of 700 to confirm the polymer identity.

### 2.6. Biomarker Analysis

To assess the potential impact of MPs and temperature on oxidative stress in *S. spinosulus* tissues, a series of biomarkers were analysed. *S. spinosulus* samples were first rinsed with distilled water and homogenized on ice at a ratio of 1:5 (*w*/*v*) in 100 mM Tris–HCl buffer (pH 7.5) containing 1 mM EDTA. Homogenization was performed using an Ultra-Turrax^®^ Disperser (IKA, Staufen, Germany). The homogenates were subsequently centrifuged at 9000× *g* for 10 min at 4 °C using a Sigma 3K30 centrifuge (Sigma GmbH, Osterode am Harz, Germany) [24]. The resulting supernatants were collected and used for biochemical analyses.

Antioxidant enzyme activities, including catalase (CAT) and superoxide dismutase (SOD), were assessed, along with measurements of total glutathione (GSH) and malondialdehyde (MDA). Lysozyme and glutathione S-transferase (GST) activities were also analysed. Except for lysozyme, enzymatic activities were measured spectrophotometrically at 25 °C using a Shimadzu UV-2100 spectrophotometer (Kyoto, Japan). CAT activity (mK/mg protein) was determined by monitoring the breakdown of H_2_O_2_ at 240 nm according to the method of Aebi (1984) [25]. SOD activity (pKat/mg protein) was evaluated at 550 nm following a previously established protocol, with cytochrome serving as the indicator [26]. Lysozyme activity was measured at 450 nm using a microplate reader (BioTek^®^ PowerWaveXS) and a bacterial suspension of *Micrococcus lysodeikticus* as a substrate [27]. GST activity (nKat/mg protein) was determined at 340 nm using GSH and 1-chloro-2,4-dinitrobenzene (CDNB) as substrates, following the method of Habig et al. (1974) [28]. GSH levels (nmol/mg protein) were quantified at 415 nm based on the protocol described by Pinya et al. (2016) [29], while MDA levels (nM/mg protein) were measured using a colorimetric assay kit (Merk Life Science S.L.U., Madrid, Spain), adhering to the manufacturer’s guidelines. Both GSH and MDA were analysed with a microplate reader (BioTek^®^ PowerWaveXS, Agilent Technologies, Madrid, Spain).

All results were normalized to the protein content of the samples, determined using the Biorad^®^ Protein Assay (Bio-Rad Laboratories, Alcobendas, Spain) with bovine serum albumin as the standard.

### 2.7. Statistical Analysis

Statistical analyses were conducted using the statistical package for social sciences (IBM PSS^®^ v29.0, Armonk, NY, USA). Data normality was assessed using the Shapiro–Wilk test. Variations in sponge health status (healthy vs. affected) across sampling periods (July, August, and November) were analysed using a two-way analysis of variance (ANOVA) and a least significant difference (LSD) post hoc test to identify significant differences between specific groups. All results are presented as mean ± standard error of the mean. A significance level of *p* < 0.05 was applied throughout the analysis.

## 3. Results

### 3.1. Water Temperature

**Figure 3:**
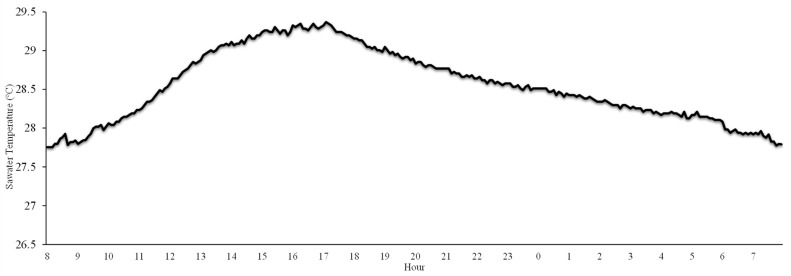
Daily temperature variation (24–25 August), showing values exceeding 29 °C for more than 5 h.

The water temperature measured at a depth of 0.8 m during *S. spinosulus* sampling was 24.1 ± 0.2 °C in July, 29.0 ± 0.1 °C in August, and 21.4 ± 0.1 °C in November. In August (between the 24th and 25th), daily temperature analysis showed a peak of 29.4 °C, with more than 5 h where the temperature exceeded 29 °C (Figure 3).

### 3.2. Characteristics of Sarcotragus spinosulus

Sponge samples collected during the initial sampling in July, which showed no signs of tissue damage, had a maximum diameter of 9.67 ± 0.54 cm and a height of 7.12 ± 0.49 cm. By August and November, sponges were differentiated into healthy and affected groups displayed diameters of 9.35 ± 0.39 cm and 9.31 ± 0.54 cm, and heights of 6.92 ± 0.33 cm and 6.75 ± 0.45 cm, respectively. Externally, affected sponges appeared duller in colour and were covered with epiphytes, while healthy sponges maintained a bright colour and were almost free of surface elements (Figure 1).

### 3.3. Presence of Vibrio spp. on Plastics and FTIR Analysis

**Figure 4:**
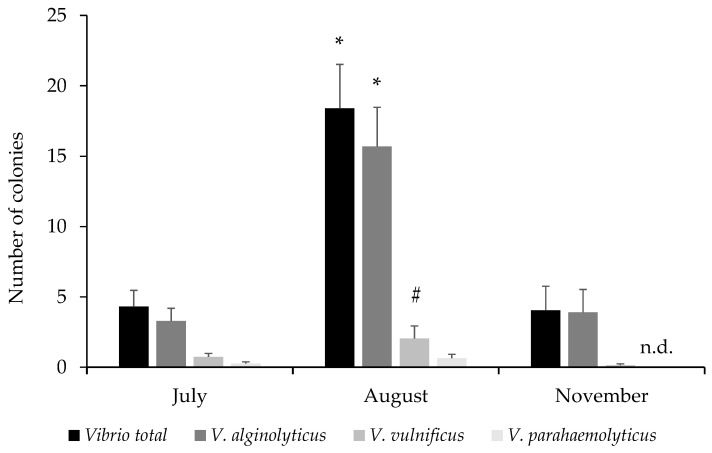
Number of *Vibrio* spp. colonies (CFU) in the plastics across the three sampling months. Note: * indicates significant differences respect July and November, and # respect to November, *p* < 0.05. n.d.—none detected.

A total of 40 plastic items collected from the area were analysed in each sampling period. The results revealed a significant increase in CFU counts in August compared to July (4.33 ± 1.14 CFU/MP in July vs. 18.40 ± 3.12 CFU/MP in August), followed by a notable decrease in November (4.05 ± 1.71 CFU/MP) (Figure 4). The values in August were statistically significant compared to the months of July and November (*p* < 0.001). Among the three analysed species, *V. alginolyticus* was predominant, accounting for over 75% of observed CFUs in all samplings. Regarding the percentage of MPs with evidence of *Vibrio* spp., 37.5% of MPs showed bacterial growth in July, rising to 77.5% in August, and then dropping to 35% in November. Specifically, a significantly higher number of colonies was observed in August compared to July and November for *Vibrio* spp. and *V. alginolyticus*; meanwhile, for *V. vulnificus*, the differences were only significant compared to November. No colonies of *V. parahaemolyticus* were observed in the plastics sampled in November.

The FTIR analysis of the 40 plastic samples from August revealed a predominance of high-density polyethylene (HDPE, 18 items, 45%), followed by polypropylene (PP, 14 items, 35%), and much smaller quantities of low-density polyethylene (LDPE, 3 items), polystyrene (PS, 3 items), and polyurethane and polyamide, with one item each. Of the 18 HDPE plastics, *Vibrio* spp. growth has been observed in 15 of them, with an average of 20.5 ± 4.8 CFU. In the case of PP, growth has been observed in 12 of the 14 items, with an average of 21.7 ± 5.8 CFU.

### 3.4. Presence of Vibrio spp. in S. spinosulus

**Figure 5:**
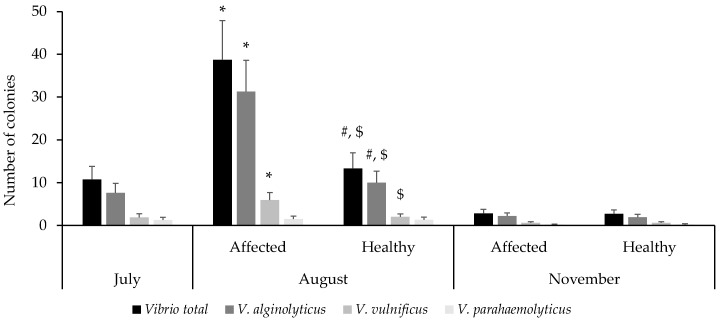
Number of *Vibrio* spp. colonies (CFU) in *Sarcotragus spinosulus* samples collected during the three sampling months, differentiated by healthy and affected specimens. Note: * indicates significant differences respect July and November; # respect to November within the same experimental group: affected or healthy; $ indicates significant differences between affected and healthy sponges, *p* < 0.05.

In *S. spinosulus*, CFU counts increased from July to August in the affected group, decreasing again in November to values similar to those of the healthy group (Figure 5). Specifically, CFU counts were 10.73 ± 3.03 in July, increasing to 38.70 ± 9.17 in the affected group in August, while remaining stable at 13.3 ± 3.62 in the healthy group. By November, CFU counts were similar between groups, with 2.80 ± 0.97 in the affected group and 2.70 ± 0.93 in the healthy group. Similar to plastics, *V. alginolyticus* was the most prevalent species, representing over 70% of CFUs. Regarding the percentage of sponges with evidence of *Vibrio* spp., bacterial growth was observed in 60% of sponges in July, 90% of the affected group and 70% of the healthy group in August, and 60% in both groups in November.

In the affected sponges, a significant increase in the number of colonies was observed in the month of August compared to July and November for *Vibrio* spp., *V. alginolyticus*, and *V. vulnificus*, while there were no differences over time in *V. parahaemolyticus*. In the case of healthy sponges, no differences were observed between the months of July and August, but lower values were observed in November compared to August for *Vibrio* spp. and *V. alginolyticus*. In August, higher values of *Vibrio* spp., *V. alginolyticus* and *V. vulnificus* were observed in the affected sponges compared to the healthy ones, which normalized in November.

### 3.5. Microplastics in S. spinosulus

**Figure 6:**
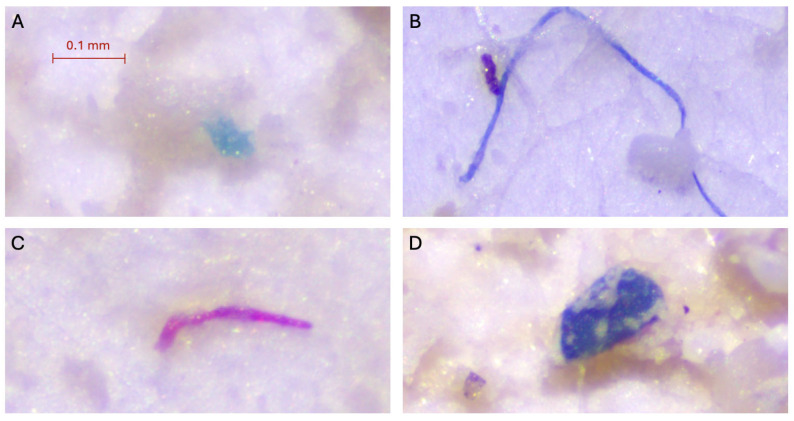
Example of images of microplastic particles found in *Sarcotragus spinosulus* at Portals Vells: (**A**) blue irregular fragment; (**B**) blue fibre; (**C**) red fibre; (**D**) blue irregular fragment.

In August, 45% of sampled sponges contained MPs, with 70% of affected individuals and 20% of healthy individuals showing items. Affected sponges had an average of 0.9 ± 0.88 MPs/sample, while healthy specimens had an average of 0.5 ± 1.08 MPs/sample. Regarding MP concentration by dry tissue weight, affected sponges contained 7.24 ± 6.63 MPs/g, significantly higher than the 1.60 ± 3.38 MPs/g found in healthy sponges. MP lengths were similar between groups, averaging 0.97 ± 0.72 mm in affected sponges and 1.21 ± 0.66 mm in healthy sponges. In terms of MP types, affected specimens contained only fibres (77.8%) and fragments (23.2%), with a similar distribution observed in healthy specimens, comprising fibres (80%) and fragments (20%). Regarding colour, the majority of items in affected sponges were blue (55.6%), followed by transparent (22.2%), whereas in healthy individuals, most items were blue (80%) (Figure 6).

### 3.6. Biomarkers in S. spinosulus

**Figure 7:**
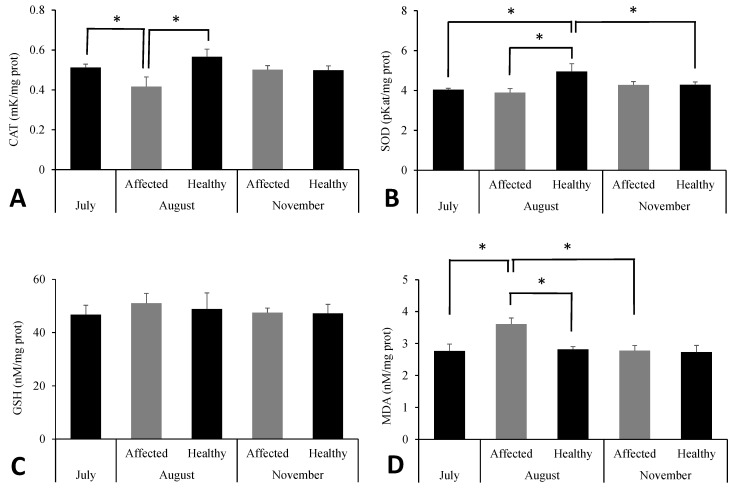
Biomarkers of oxidative stress—(**A**) catalase and (**B**) superoxide dismutase (SOD) activities, (**C**) glutathione levels (GSH), and (**D**) malondialdehyde levels (MDA)—in *Sarcotragus spinosulus* in the three sampling periods. * Significant differences between the involved groups at *p* < 0.05.

Biomarkers related to oxidative stress are presented in Figure 7. An increase in CAT and SOD enzyme activities was observed in healthy sponges in August compared to July (CAT: *p* = 0.038; SOD: *p* = 0.005) and the affected group (CAT: *p* = 0.002; SOD: *p* = 0.001). For SOD, significant differences were also noted between August and November (*p* = 0.036). GSH levels remained stable across groups and sampling times (*p* > 0.05). MDA levels were statistically higher in the affected group in August compared to all other groups (*p* < 0.01).

**Figure 8:**
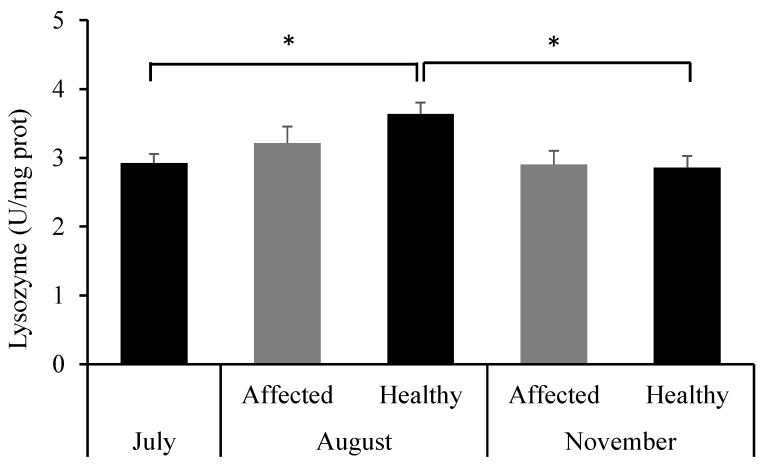
Activity of lysozyme in *Sarcotragus spinosulus* in the three sampling periods. * Significant differences between the involved groups at *p* < 0.05.

The antibacterial enzyme lysozyme showed a significant increase in activity in the healthy group in August compared to July (*p* = 0.009) and November (*p* = 0.005), whereas differences in the affected group were not statistically significant (Figure 8).

**Figure 9:**
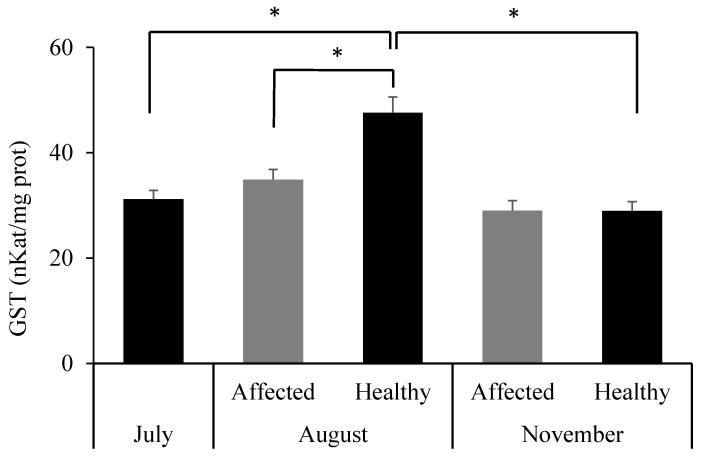
Activity of glutathione s-transferase (GST) in *Sarcotragus spinosulus* in the three sampling periods. * Significant differences between the involved groups at *p* < 0.05.

No significant differences were observed between groups in August (*p* = 0.114). Detoxification enzyme glutathione S-transferase activity increased significantly in the healthy group in August compared to July (*p* < 0.001) and November (*p* = 0.001), with significant differences between groups in August (*p* = 0.001) (Figure 9).

## 4. Discussion

The present study provides important insights into the effects of plastics as carriers of *Vibrio* spp. and increasing temperatures on oxidative stress, detoxification, and immune response in marine sponges. The findings reveal a complex interplay between environmental stressors and host physiological responses, emphasizing the vulnerability of *S. spinosulus* to anthropogenic pressures. In August, when temperatures are higher, it has been observed that sponge specimens with an affected appearance exhibit less active protection mechanisms compared to those with a healthy appearance, along with an increased presence of *Vibrio* spp., which could even compromise their viability. Once temperatures decrease, the appearance of the affected sponges normalizes, as does the number of bacterial colonies, highlighting the importance of temperature in these coastal environments.

In fact, one of the major challenges faced by marine species, particularly sessile benthic organisms living in shallow areas, is the progressive increase in water temperature driven by human activity. Rising temperatures are particularly evident in regions that are highly sensitive to climate variability and global warming, such as the Mediterranean Sea [23]. Since 1982, the Mediterranean’s sea surface temperature has been increasing at a rate of approximately ~0.4 °C per decade [30], and this trend is expected to intensify throughout this century [31]. This phenomenon is also linked to marine heatwaves, defined as prolonged periods of extremely high sea surface temperatures, which are becoming longer and more frequent [32]. The present study has demonstrated that, in August, sea surface temperatures can exceed 29 °C, posing a significant risk to marine sponges. The metabolic stress experienced by sponges may impair their ability to cope with new stressors, such as bacterial overgrowth [20]. In this context, it has been hypothesized that even bacterial species usually associated with sponges may become harmful if the temperature increases excessively [20].

In addition to elevated temperature conditions, the presence of thermos-dependent pathogens can contribute to the weakening or even death of certain organisms. Increased seawater temperatures promote pathogen virulence and/or enhance host susceptibility [33]. Specifically, the spread of *Vibrio*-related diseases has been identified as an emerging global concern linked to rising seawater temperatures [34]. In this regard, *Vibrio* spp. have also been associated with the onset of diseases in the genus *Sarcotragus* in the Mediterranean Sea [35]. Consistent with these previous findings, the present study has shown an increase in the presence of *Vibrio* spp. with rising seawater temperatures, a trend that is particularly pronounced in *S. spinosulus* specimens with a damaged appearance. Similar results have been observed during high-temperature events, with a higher presence of *Vibrio* spp. in organisms exhibiting tissue lesions across various species, including the sponge *S. fasciculatus* and corals like *Oculina patagonica*, *Phyllangia mouchezii*, and *Balanophyllia europaea* [21,36]. A massive mortality event of *Sarcotragus foetidus* that occurred in September 2021 in the Aegean Sea (eastern Mediterranean) was attributed to prolonged elevated temperatures, which promoted the proliferation of pathogenic *Vibrio* species [37]. These conditions triggered or exacerbated the progression of diseases in sponge.

Plastics are becoming an increasingly significant problem due to their high and growing presence in the marine environment, leading to associated issues such as entanglement and ingestion in marine fauna. In the present study, the presence of MPs was observed in the tissues of *S. spinosulus* sampled in August. These findings align with those reported by Krikech et al. (2023) [38], who demonstrated the presence of MPs in various sponge species, including *S. spinosulus*, along the Moroccan Mediterranean coast. Interestingly, the presence of MPs was higher in sponges with an affected appearance compared to healthy ones. This difference may stem from the existence of resistance or expulsion mechanisms in sponges, which enable them to thrive even in contaminated waters [39]. Specimens showing evidence of damage may be weakened and lack the same capacity as healthy individuals to expel foreign particles. Consistently, it has been shown that the ingestion of MPs can reduce filtration and respiration rates, which would result in lower energy production and diminished capacity to respond to stressors [40]. This could further explain the lack of activation of antioxidant, detoxification, and immune defence mechanisms observed in the affected specimens.

Another aspect related to plastics is their polymeric nature, which allows for the adsorption of substances on their surface, posing a potential toxicological risk to the health of exposed organisms [41]. Among the substances that can be adsorbed are various types of chemical pollutants, such as persistent organic pollutants and heavy metals. Additionally, plastics can serve as a habitat for numerous forms of life, such as bacteria [10]. Furthermore, since plastics are relatively new and artificial substrates in marine systems, they can disrupt natural boundaries and interfere with ecosystem functioning [42]. In the present study, the growth of *Vibrio* spp. was evidenced on a large number of plastics collected throughout the study, with a notable increase at higher seawater temperatures. These plastics can thus act as carriers and even accumulators of bacteria, in this case, of the *Vibrio* genus, which may subsequently interact with diverse species and pose a risk to their health. The sponge *S. spinosulus*, due to its high filtering capacity, can capture MPs, as also evidenced in this study, and consequently be exposed to compounds or organisms adhered to them. When combined with elevated temperatures, which can weaken some specimens, this may significantly impact their physiological functions and even contribute to their death.

The potential effects of elevated temperatures and the presence of *Vibrio* spp. on *S. spinosulus* were evaluated through the analysis of various biomarkers. Regardless of their function—antioxidant, detoxification, or immune-related—the response pattern was very similar, with an increase observed in August in healthy specimens, whereas affected specimens failed to exhibit this response. Once the water temperature and exposure to plastics with a lower bacterial load decreased, the parameters of the sponges from both groups, affected and healthy, become similar. Regarding oxidative stress markers, an increase in CAT and SOD activity was observed in the healthy group during August, with no evidence of oxidative damage as assessed by MDA levels. In contrast, the affected group did not show this increase in enzymatic activities but exhibited a rise in MDA levels, indicative of a pro-oxidative and stressful state. The rise in temperature leads to an increase in metabolic activity and, consequently, the production of reactive oxygen species [43]. Furthermore, the additional exposure to toxic substances such as plastics and bacteria in August highlights the critical need for sponges to activate their antioxidant defence mechanisms. Various studies have demonstrated an antioxidant response to thermal stress in different sponges under controlled conditions, showing that at elevated temperatures, a lack of response may occur, potentially compromising sponge survival [44,45].

GST, a phase II detoxification enzyme, also increases under stress conditions such as elevated temperatures or the presence of toxic substances [45,46]. In the present study, the increase observed in August, particularly in sponges with a healthy appearance, may result from the combined action of temperature and greater exposure to contaminants such as MPs. In a previous study, higher GST activity was observed in *S. spinosulus* specimens collected from a contaminated area compared to those collected from a less human-impacted zone [47]. Lysozyme activity also increased in August in the healthy group in response to a higher bacterial load. Similarly, an increase in lysozyme production has been observed following exposure to peptidoglycans of bacterial origin [48].

The results of this study provide a foundation for future research into the toxicity of plastic-related byproducts on marine organisms, particularly regarding leachates from tire particles and biodegradable plastics [49,50]. Recent studies have demonstrated that these leachates can cause intergenerational effects, including impaired growth and reduced reproductive capacity, with the toxicity of biodegradable plastics on aquatic organisms remaining a significant challenge [49,50,51]. Furthermore, *S. spinosulus* is particularly vulnerable to foreign particles such as MPs and nanoplastics (<1 µm), as well as dissolved or suspended contaminants, due to its high clearance rate, as it is capable of filtering a water volume 17 times its own within just one hour [19]. Nanoplastics are an increasing concern for aquatic organisms, particularly due to their potential to unintentionally enter organisms via carrier particles and their significant toxic effects [52]. This vulnerability is particularly concerning given that previous research by Saliu et al. (2022) found that 70% of studied sponges contained plastic particles within the MP and nanoplastics size range [53].

## 5. Conclusions

This study provided evidence of the multifaceted impact of elevated seawater temperatures and plastic pollution on the physiological responses of *S. spinosulus*. The findings demonstrate that healthy sponge specimens exhibit an adaptive increase in antioxidant-, detoxification-, and immune-related biomarkers during periods of elevated stress. Conversely, affected specimens show a lack of such responses, accompanied by oxidative damage and increased bacterial colonization, particularly by *Vibrio* spp. This study highlights the critical role of temperature in shaping sponge resilience and the additional risks posed by plastics acting as vectors for harmful bacteria. Further studies are needed to investigate the long-term impacts of chronic thermal stress and MP exposure on sponge populations, with a particular focus on their capacity for recovery during cooler periods.

## Data Availability

Researchers wishing to access the data used in this study can make a request to the corresponding author: antoni.sureda@uib.es.
